# Persistent Activation of the Innate Immune Response in Adult *Drosophila* Following Radiation Exposure During Larval Development

**DOI:** 10.1534/g3.115.021782

**Published:** 2015-09-01

**Authors:** Lisa J. Sudmeier, Sai-Suma Samudrala, Steven P. Howard, Barry Ganetzky

**Affiliations:** * Laboratory of Genetics, University of Wisconsin, Madison, Wisconsin 53706; † Department of Human Oncology, University of Wisconsin School of Medicine and Public Health, Madison, Wisconsin 53792

**Keywords:** innate immunity, neuroinflammation, radioprotection, radiosensitivity, genetics of immunity

## Abstract

Cranial radiation therapy (CRT) is an effective treatment for pediatric central nervous system malignancies, but survivors often suffer from neurological and neurocognitive side effects that occur many years after radiation exposure. Although the biological mechanisms underlying these deleterious side effects are incompletely understood, radiation exposure triggers an acute inflammatory response that may evolve into chronic inflammation, offering one avenue of investigation. Recently, we developed a *Drosophila* model of the neurotoxic side effects of radiation exposure. Here we use this model to investigate the role of the innate immune system in response to radiation exposure. We show that the innate immune response and NF-ĸB target gene expression is activated in the adult *Drosophila* brain following radiation exposure during larval development, and that this response is sustained in adult flies weeks after radiation exposure. We also present preliminary data suggesting that innate immunity is radioprotective during *Drosophila* development. Together our data suggest that activation of the innate immune response may be beneficial initially for survival following radiation exposure but result in long-term deleterious consequences, with chronic inflammation leading to impaired neuronal function and viability at later stages. This work lays the foundation for future studies of how the innate immune response is triggered by radiation exposure and its role in mediating the biological responses to radiation. These studies may facilitate the development of strategies to reduce the deleterious side effects of CRT.

Radiation therapy is an integral component of cancer treatment regimens. Radiation acts by inducing DNA damage and the generation of reactive oxygen species in cancer cells, which together promote cell death. Although radiation is mostly targeted to the tumor or resection bed during therapy, healthy cells are also unavoidably irradiated . Consequently, healthy tissue damage is a side effect of radiation therapy. For example, cognitive deficits resulting from injury to healthy brain cells is a common side effect of cranial radiation therapy for brain tumors (Armstrong *et al.* 2009; Ellenberg *et al.* 2009; Mulhern *et al.* 2005; Packer *et al.* 2003; Redmond *et al.* 2013). As cancer survival rates increase, there is growing attention on reducing deleterious side effects of therapy. Moreover, developing strategies to protect healthy tissue from radiation damage may allow for the use of higher doses to increase the tumor-killing efficacy of radiation. Developing therapies for protecting healthy tissue from damage will require a better understanding of the biological pathways activated by radiation. Some of these pathways may function as endogenous radioprotectors, and others may promote radiation-induced damage. Dissecting the response of healthy tissue to radiation exposure will aid in the development of pharmacological agents to selectively promote radiation protection or mitigate the side effects of radiation exposure.

Many studies investigating radioprotective compounds have focused on antioxidants and synthetic thiols (Weiss and Landauer 2003). However, anti-inflammatory drugs may be the most effective agents for reducing damage to healthy tissue from radiation exposure (Moding *et al.* 2013). A number of mammalian models of radiation-induced damage have demonstrated increased cytokine expression following radiation exposure (Rubin *et al.* 1995; Hong *et al.* 1995; Chang *et al.* 2011). Moreover, increased NF-ĸB activation is seen in the brain and other tissues, including liver and intestine, following radiation exposure (Chang *et al.* 2011; Wang *et al.* 2004; Zhou *et al.* 1999; Raju *et al.* 1998). The level of NF-ĸB activated by radiation varies between organs and with time since exposure and causes organ-specific changes in gene expression profiles (Chang *et al.* 2011).

Radiation-induced inflammation can have varied effects. Persistent inflammation in lung tissue after radiation exposure is associated with increased collagen production and pulmonary fibrosis (Rubin *et al.* 1995). Moreover, sustained activation of astrocytes and microglia months after radiation exposure reveals persistent reactive gliosis (Chiang *et al.* 1993). Despite the negative effects of chronic inflammation, NF-ĸB is activated by DNA damage (Miyamoto 2011) and may have an acute radioprotective role. Reduced NF-ĸB activity in mice increases radiation sensitivity and results in greater lethality from whole-body radiation exposure (Wang *et al.* 2004), consistent with a radioprotective role for NF-ĸB. Conversely, promoting NF-ĸB activation using Toll-like receptor (TLR) agonists increases radioprotection both in mouse and primate models (Burdelya *et al.* 2008; Hu *et al.* 2013; Shakhov *et al.* 2012). Treating mice with the TLR5 agonist bacterial flagellin before total body radiation exposure reduces mortality and promotes maintenance of normal stem cell proliferation levels in the small intestine and reduced apoptosis in hematopoietic and intestinal cells (Burdelya *et al.* 2008).

The radioprotective benefits of TLR receptor agonists suggest that the immune system may also be a promising target for reducing radiation toxicity following cranial radiation therapy (CRT). Studies using mammalian models have demonstrated that inflammatory pathways are activated in the brain following radiation exposure (Chang *et al.* 2011; Raju *et al.* 1998; Hong *et al.* 1995), and that activation of microglia and astrocytes in the brain leads to gliosis months after radiation exposure (Chiang *et al.* 1993). Nonetheless, many questions about the postradiation inflammatory response in the brain remain unanswered, and little is known about the inflammatory consequences of irradiating a developing brain. Moreover, a better understanding of the time course of activation of inflammatory pathways, especially in the brain, will be critical for predicting when specific pharmacological agents could be most beneficial. For example, there is a radioprotective benefit achieved with TLR agonists administered within 24 hr before or 1 hr after irradiation (Burdelya *et al.* 2008), but another therapeutic strategy could be to target the inflammation that persists after radiation exposure.

Pediatric brain tumor patients in particular would benefit from more effective radioprotective strategies following CRT to treat pediatric central nervous system malignancies. Although cure rates are now more than 70%, these patients suffer long-term neurological side effects, including cognitive impairments, motor and coordination deficits, and increased susceptibility to seizure disorders (Armstrong *et al.* 2009; Edelstein *et al.* 2011b; Ellenberg *et al.* 2009; Mulhern *et al.* 2005; Packer *et al.* 2003; Redmond *et al.* 2013). This population deserves specific attention in the development of radioprotective agents because pediatric and adult brains differ markedly with the former undergoing dynamic alterations in synaptic density and metabolism throughout development (Huttenlocher 2002). Consequently, it is not surprising that younger patients undergoing CRT suffer more severe long-term side effects than older patients who receive the same treatment (Edelstein *et al.* 2011b; Mulhern *et al.* 2005).

We recently developed a *Drosophila* model of the long-term neurotoxic side effects of radiation exposure during development (Sudmeier *et al.* 2015). This model demonstrates that in flies, as in human patients who undergo CRT for pediatric malignancies, there are long-term effects in the adult, such as impaired neurological function and cellular changes in the brain. A key advantage of a fly model is that we can use genetic tools to dissect the biological pathways that confer radioprotection or radiosensitivity during development. Flies, like other invertebrates, have innate immunity but lack an adaptive immune system (Bulet *et al.* 2004). The *Drosophila* innate immune system, which is conserved with that of mammals, consists primarily of the Toll and Imd pathways, which together combat fungal and bacterial infections. The canonical Toll signaling pathway consists of the cytokine Spätzle, the Toll membrane receptor, the adapters MyD88 and Tube, the Pelle kinase, Cactus, which is homologous to IĸB, and the transcription factors Dorsal and Dif (Belvin and Anderson 1996; Tauszig-Delamasure *et al.* 2002; Valanne *et al.* 2011). The canonical Imd pathway consists of the transmembrane receptor PCRP-LC, TAK1 (the mitogen-activated protein 3 kinase), an inhibitor of apoptosis protein (DIAP2), IKKβ/ird5, IKKγ/Kenny (the *Drosophila* form of the IKK signalosome), dFADD, the caspase Dredd, and the transcription factor Relish (Myllymäki *et al.* 2014). Activation of either pathway results in activation of NF-ĸB transcription factors (Dorsal and Dif in the Toll pathway, Relish in the Imd pathway), which translocate to the nucleus and induce expression of antimicrobial peptides (AMPs). AMPs mediate the humoral component of *Drosophila* innate immunity (Lemaitre and Hoffmann 2007). Chronic activation of the innate immune system in neurons and glia of adult flies via overexpression of AMPs is sufficient to promote neurodegeneration (Cao *et al.* 2013; Petersen *et al.* 2012).

Here, we use our fly model to investigate the effect of radiation exposure during larval development on the subsequent induction and persistence of the innate immune response. Our data indicate that there is sustained activation of the innate immune response in adult flies that were exposed to radiation during larval development. Moreover, we demonstrate that neurons and glia in the adult brain mount an immune response following larval irradiation. Although activation of the innate immune response may promote survival from the initial radiation exposure, persistent activation of the innate immune system may contribute to the subsequent neurological impairments observed in adult flies weeks after radiation exposure.

## Materials and Methods

### *Drosophila* strains and culture

Flies were maintained on standard cornmeal-molasses medium at 25°. Canton-S was used for qRT-PCR experiments. *Metchnikowin*::*GFP* and *AttacinA*::*GFP* stocks (provided by David Wassarman, University of Wisconsin-Madison and Ylva Engstrom, Stockholm University, Stockholm, Sweden) were used to visualize AMP expression in the brain. *Rel^E20^ spz^4^* and *Rel^E20^* were obtained from the Bloomington Stock Center. *Spz^4^* was provided by Laura Johnston, Columbia University.

### Irradiation of late third instar larvae

Larval irradiation was performed as described in Sudmeier *et al.* (2015). Briefly, wandering third instar larvae were collected, counted, and transferred to fresh culture vials (30–50 larvae per vial). These vials were irradiated using a ^137^Cs irradiator with an average dose rate of 6.5 Gy/min.

### Quantitative Real Time-PCR (qRT-PCR)

**Figure 1:**
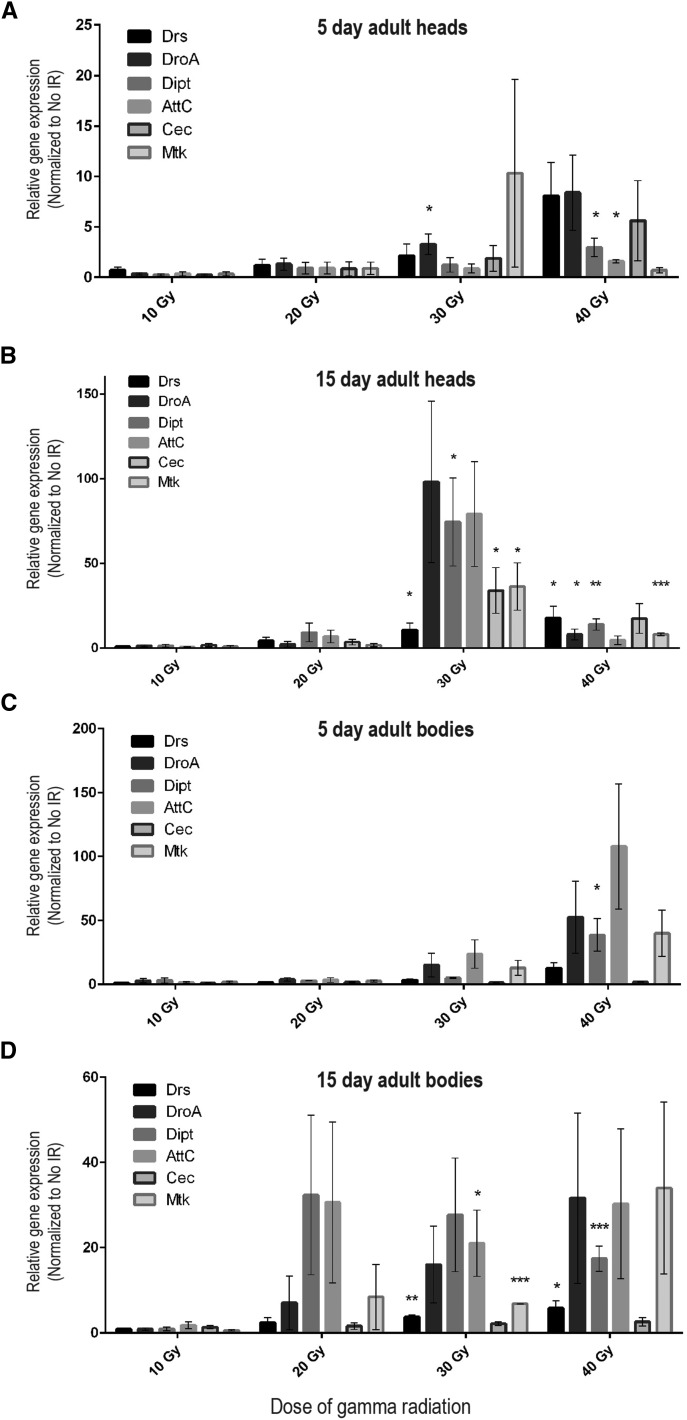
AMP expression in adult heads and bodies is increased following irradiation of larvae. For each dose of radiation during the late third instar, expression of mRNAs encoding six different AMPs in heads (A, B) and bodies (C, D) of 5-d-old and 15-d-old adults was measured by qRT-PCR analysis. Wild-type male *Canton-S* flies were used for these experiments. Bars represent mean expression of each AMP mRNA (3–5 trials each) normalized to mean expression of the corresponding RNA in nonirradiated, age-matched controls (fold-induction). Error bars are SEM. *Rp49* was used as the reference gene. See Table S1 for primers used. *P < 0.05, **P < 0.05, and ***P < 0.01 based on Student’s *t*-test comparing relative expression in 40 Gy or 30 Gy samples to 10 Gy samples.

For experiments in Figure 1, adult flies were allowed to mate for 2 d following eclosion and then collected, sorted by sex, and transferred to fresh vials (≤15 males or females per vial) containing standard medium and aged at 25°. Flies were transferred to fresh food every 3 d. On day 5 or 15 of adulthood, flies were anesthetized with CO_2_, a razor blade was used to cut heads from bodies, and samples were transferred to microcentrifuge tubes and frozen at −80°. For experiments in Supporting Information, Table S2, whole pupae were transferred to microcentrifuge tubes and frozen at −80° 4–4.5 hr after they were irradiated as late third instar larvae. RNA was extracted from frozen samples using TRI Reagent RT (Molecular Research Center, Cincinnati, OH). The iScript cDNA Synthesis Kit (Bio-Rad, Hercules, CA) was used to generate cDNA. Quantitative real-time PCR was performed using SYBR Green Supermix (Bio-Rad, Hercules, CA) according to the manufacturer instructions. Primer sequences are provided in Table S1. *Rp49* was used as the reference gene. PCR was performed with a Bio-Rad iCycler and the following program: 35 cycles: step 1: 95° for 10 sec; step 2: 60° for 30 sec; step 3: 72° for 40 sec each cycle. Males and females both demonstrated a dose-dependent increase in AMP expression at 5 and 15 d in heads and bodies, but only data for males are presented here.

### Immunohistochemistry and microscopy

Brains were dissected, stained, and imaged as described in Sudmeier *et al.* (2015). The antibodies used are as follows: Chicken anti-Green Fluorescent Protein (Life Technologies, Carlsbad, CA; 1:500), Mouse anti-Repo (Developmental Studies Hybridoma Bank, University of Iowa, Iowa City, IA; 8D12, 1:50), Rat anti-Elav (Developmental Studies Hybridoma Bank, University of Iowa, Iowa City, IA; 7E8A10, 1:250), DAPI (Sigma-Aldrich, St. Louis, MO; 1 mg/L), Goat anti-Chicken Alexa Fluor-488 (Life Technologies, Carlsbad, CA; 1:200), Goat anti-Mouse Alexa Fluor-568 (Life Technologies, Carlsbad, CA; 1:200), and Goat anti-Rat Alexa Fluor-633 (Invitrogen, Carlsbad, CA; 1:200).

### Statistical analyses

GraphPad Prism (GraphPad Software, San Diego, CA) was used for Student’s *t*-test analyses of eclosion and AMP transcription levels. GraphPad outlier calculator was used to identify outliers in qPCR data (http://graphpad.com/quickcalcs/Grubbs1.cfm).

### Data availability

Table S1 contains primer sequences for all qPCR primers used in experiments presented here. Table S2 contains qPCR data for AMP expression levels 4–4.5 hr after larval irradiation. Stains used in these experiments available upon request.

## Results

### Activation of the innate immune response by radiation exposure during larval development persists in adults

Studies in both mouse and humans demonstrate that radiation exposure can trigger persistent inflammation (Neriishi *et al.* 2001; Hayashi *et al.* 2005; Hayashi *et al.* 2012; Chiang *et al.* 1993; Rubin *et al.* 1995). One of our goals in establishing a *Drosophila* model of radiation exposure during larval development was to investigate the mechanisms contributing to deleterious consequences in adults long after radiation exposure ends because understanding these mechanisms could facilitate new approaches for therapeutic intervention following CRT. In particular, we have focused on the neurological consequences in adults following larval irradiation (Sudmeier *et al.* 2015). Consistent with the long-term neurological impairments observed in pediatric CRT patients, including motor deficits, premature aging, and loss of white matter integrity (Redmond *et al.* 2013; Edelstein *et al.* 2011a; Schuitema *et al.* 2013), we observed impaired motor behavior, shortened lifespan, and increased neurodegeneration in adult flies exposed to radiation during larval development. Because sustained activation of the innate immune response in the nervous system promotes neurodegeneration in *Drosophila* (Cao *et al.* 2013, Petersen *et al.* 2012), we hypothesized that radiation exposure during larval development leads to persistent inflammation in the brains of adult flies, comparable to observations in mice and humans (Neriishi *et al.* 2001; Hayashi *et al.* 2005; Chiang *et al.* 1993).

To measure activation of the innate immune response, we used quantitative real-time PCR (qRT-PCR) to measure antimicrobial peptide (AMP) transcript levels in heads of adult flies following irradiation during larval development. Toll and Imd signaling lead to activation of NF-ĸB transcription factors, which positively regulate expression of AMPs (Lemaitre and Hoffmann 2007). We observed an increase in AMP expression in heads of 5-d-old adults that were irradiated 10 d earlier, during the late third larval instar, compared with age-matched, nonirradiated controls (Figure 1A). We performed a similar analysis on 15-d-old adults that had been irradiated 20 d earlier and also observed an increase in AMP transcripts compared with age-matched, nonirradiated controls, indicating that activation of the immune response persists at least 3 wk after radiation exposure (Figure 1B). Furthermore, the level of increased AMP expression in 15-d-old adults appears even greater than that observed in 5-d-old adults, suggesting that activation of the innate immune response following radiation exposure continues to increase as the adults age. Together, these results indicate that the innate immune system is activated by radiation exposure during development and remains activated in adulthood weeks after irradiation and may further increase over time. We propose that this sustained inflammatory response contributes to the neurological deficits we observe in adults following larval irradiation (Sudmeier *et al.* 2015).

To determine whether the radiation-induced activation of the innate immune response is specific for the nervous system or is also present in other adult tissues, we used qRT-PCR to measure AMP transcript levels in bodies of adult flies that were irradiated during larval development. Similar to the trend observed in adult heads following radiation exposure during development, AMP expression is increased in the bodies of both 5-d-old and 15-d-old adult flies that were irradiated during the late third larval instar (Figure 1, C and D). Although we did not dissect this response at the level of individual tissues, these results indicate that there is persistent systemic activation of AMP expression in adult flies following radiation exposure during development.

### AMP expression is increased in adult brain following radiation exposure during larval development

**Figure 2:**
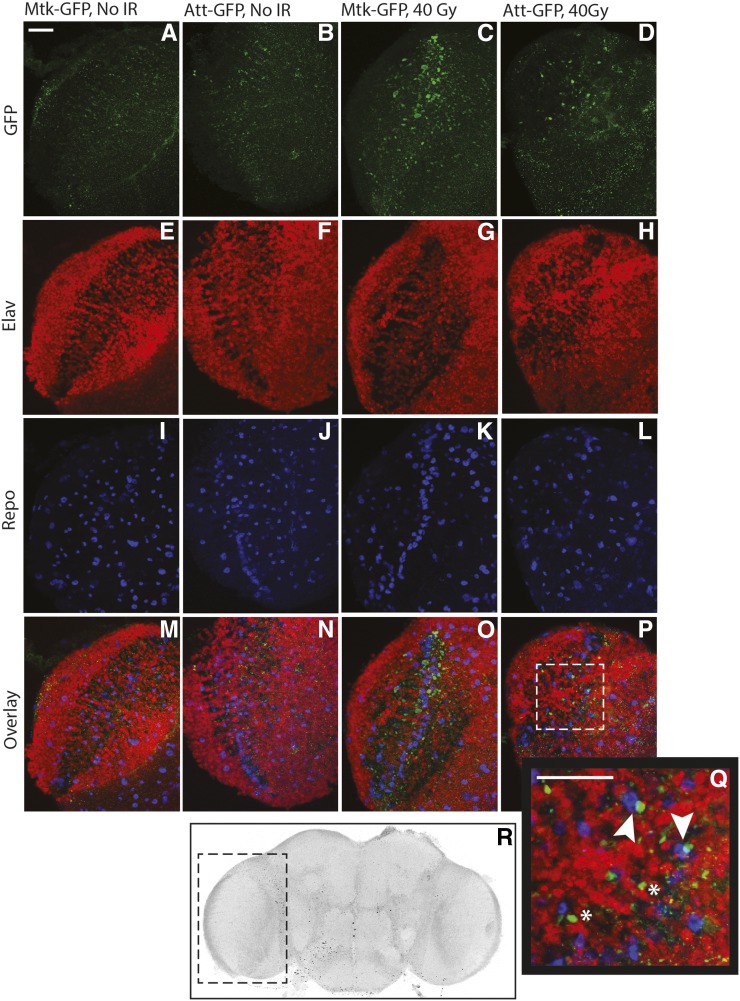
AMP-GFP reporter expression is increased in adult brains following irradiation of larvae. Following exposure of late third instar larvae to a 40-Gy dose of radiation, surviving adults were aged for 5 d after eclosion and brains were dissected for confocal microscopy. (A–Q) Confocal stacks are shown of *Metchnikowin-GFP* and *Attacin-GFP* brains immunostained for GFP (green, A–D), the neuronal (nuclear) marker Elav (red, E–H), and the glial (nuclear) marker Repo (blue, I–L). All markers are shown in the overlay (M–P). Images are of the optic lobe, as shown by the dashed box in (R). The indicated region of (P) (*Attacin-GFP*) is shown at higher magnification in the inset (Q). Brains from adults that were irradiated during larval development show increased GFP expression (C, D) compared to brains from adults that were not irradiated as larvae (A, B). Some of the GFP+ puncta are in close proximity to nuclei that are Repo+ (arrowheads in Q) or Elav+ (* in Q). Scale bars are 20 μm; 7–10 brains of each genotype for each condition were analyzed.

In response to invading pathogens, AMP expression and secretion is induced primarily in the fat body, the fly equivalent of the mammalian liver (Lemaitre and Hoffmann 2007). Although most of the fat body is located in the abdomen, the *Drosophila* head also contains fat body tissue. Our qRT-PCR experiments were performed on whole adult heads, which could reflect radiation-induced changes in AMP expression in brain, fat body, or both tissues. Therefore, we used AMP-GFP reporter lines to visualize the pattern of AMP expression in brains following radiation exposure during development. These lines express GFP under the control of AMP promoters (Tzou *et al.* 2000). In 5-d-old adults that were exposed to radiation during the late third larval instar, we observe increased GFP expression in dissected brains, both in neurons and glia based on proximity of GFP+ puncta to nuclear neuronal and glial markers (Figure 2). This result demonstrates that the innate immune system is activated in brains following irradiation during development and remains activated for at least 10 d after exposure. This result is consistent with the increase in AMP transcripts we observe in adult heads following larval irradiation. We suspect that activation of the innate immune response also occurs in fat body cells in the head, but we have not specifically measured this response.

Because overexpression of AMPs in *Drosophila* glia or neurons is sufficient to promote neurodegeneration (Cao *et al.* 2013; Petersen *et al.* 2012), we propose a model in which the innate immune system is activated following radiation exposure during development and remains activated both in brain and in other adult tissues weeks after irradiation. This persistent inflammation likely contributes to the adult neurological deficits and premature aging associated with radiation exposure during development.

### Activation of innate immunity may promote survival of larvae following radiation exposure

**Figure 3:**
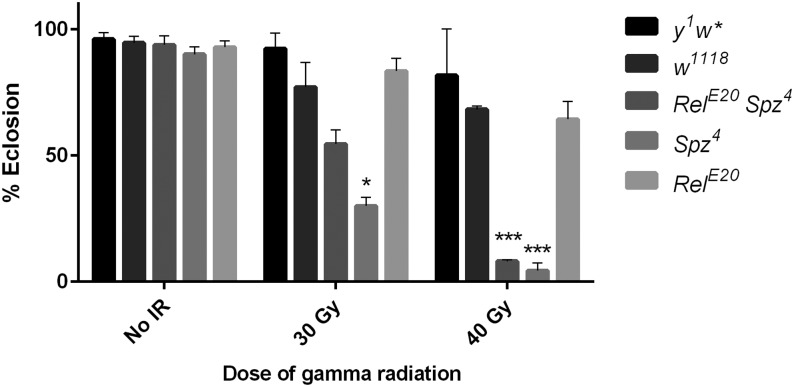
Activation of the innate immune response may be radioprotective during larval development. For each dose of radiation, the percentage of irradiated third instar larvae that complete development and eclose as adults is shown for the indicated genotypes. Loss of both Toll and Imd signaling (*Rel^E20^ spz^4^*) or Toll signaling alone (*spz^4^*) results in increased sensitivity to radiation compared with controls. Each bar represents mean ± SEM for two to five trials with 20–40 larvae per trial. ***P < 0.001 based on Student’s *t*-test comparing *Rel^E20^ spz^4^* 40 Gy or *spz^4^* 40 Gy to *w^1118^* 40 Gy. *P < 0.05 based on Student’s *t*-test comparing *spz^4^* 30 Gy to *w^1118^* 30 Gy.

The persistent neuroinflammatory response triggered by exposing larvae to radiation with its potential long-term deleterious neurological consequences raises the question of why such a disadvantageous response would be activated to begin with. One possibility is suggested by work in adult mouse and primate models demonstrating that activation of Toll-like receptor 5 confers radioprotection (Burdelya *et al.* 2008). Possibly, activation of the innate immune response following radiation exposure confers a selective advantage by somehow enabling organisms to survive the immediate consequence of this exposure. The long-term deleterious consequences that ultimately ensue would then result from subsequent dysregulation of the innate immune response and failure to properly downregulate the response over time. To examine the possibility that innate immunity is initially radioprotective in *Drosophila*, we irradiated *Relish spatzle* (*Rel spz*) double mutant late third instar larvae and quantified survival to eclosion as described previously (Sudmeier *et al.* 2015). *Rel spz* mutants are deficient in both the Imd and Toll signaling pathways. Relish is the transcription factor activated by the Imd pathway, and Spatzle is the ligand for the Toll receptor. Although unirradiated double mutants have nearly normal viability, survival of *Rel spz* larvae through eclosion is substantially reduced following irradiation (Figure 3). Compared with wild-type controls, eclosion of *Rel spz* larvae is reduced by 20% and 60% following exposure to radiation doses of 30 Gy and 40 Gy, respectively (Figure 3). This result suggests that the innate immune response promotes survival following radiation-induced damage during development.

*Rel spz* double mutants are deficient for both Imd- and Toll-mediated immunity. Thus, sensitivity to radiation during development of these larvae may be due either to a generally impaired immune response or to a more specific loss of the Imd pathway or the Toll pathway. To examine these possibilities, we irradiated single *Rel* and *Spz* mutants and quantified survival to eclosion. We used the *Rel^E20^* and *Spz^4^* alleles, the same alleles present in the *Rel spz* double mutant. Both mutants have nearly normal viability in the absence of radiation. Following irradiation, survival of *Rel* mutants is comparable to genetic background control strains (Figure 3). However, survival of *spz* mutants to eclosion is substantially reduced following irradiation and is comparable to that of *Rel spz* mutants (Figure 3). Together, these preliminary data suggest that the Toll pathway plays a predominant role over the Imd pathway in conferring radioprotection during *Drosophila* development. Nonetheless, further experiments are necessary to confirm these results, including tests of the radiation sensitivity of additional *Rel* and *spz* alleles as well as mutations affecting other components of the Toll and Imd pathways.

The results above demonstrate that larvae with an impaired innate immune response are less likely to survive to adulthood following exposure to radiation compared with normal larvae. To begin to examine how soon after irradiation the innate immune response is activated, we measured AMP transcript levels by qRT-PCR in pupae 4–4.5 hr after irradiating larvae during the late third instar. At this time point, we did not yet observe a significant increase in AMP expression (Table S2). The irradiated larvae progress through metamorphosis for 5 d before eclosing as adults. We infer that there is a critical period sometime later than 4–4.5 hr after irradiation and prior to eclosion during which activation of the innate immune response is required to promote survival to adulthood. It will be necessary to perform a more complete developmental time course of AMP expression following irradiation to determine when activation of the innate immune response begins. This information could provide important insights about what triggers activation following irradiation and how this activation promotes survival through eclosion.

## Discussion

We have shown that following irradiation of third instar larvae, the innate immune response is activated in adult brains and remains activated up to 3 wk after exposure. This persistent neuroinflammation likely contributes to the subsequent long-term neurological deficits observed in these flies. In contrast with the deleterious long-term effects associated with persistent neuroinflammation, our preliminary results suggest that activation of the innate immune response may confer short-term benefits that enable irradiated larvae to survive through eclosion. The potential double-edged effect of the innate immune response following radiation exposure will likely have to be taken into consideration in the development of any potential radioprotective strategies targeting the innate immune response so that treatment can be timed properly.

Although our data demonstrate that irradiating larvae leads to persistent activation of the innate immune response in the brain, we still do not know the mechanism. This activation could be a direct consequence of effects of radiation on the nervous system. Alternatively, the elevated immune response could be secondary to septicemia resulting from radiation-associated damage to the intestinal barrier and resultant leakage of bacteria into the hemolymph (Mettler and Voelz 2002). Previous studies in mammals demonstrated that radiation exposure and DNA damage can activate NF-ĸB (Chang *et al.* 2011; Miyamoto 2011; Wang *et al.* 2004; Zhou *et al.* 1999; Raju *et al.* 1998). These results are consistent with activation of the innate immune response via release of damage-associated molecular pattern molecules (DAMPs) from neurons damaged by radiation exposure (Jounai *et al.* 2012). The radioprotective effects conferred by activation of the Toll pathway in mammals (Burdelya *et al.* 2008; Hu *et al.* 2013; Shakhov *et al.* 2012) together with our preliminary data suggesting a radioprotective role for Toll in *Drosophila* are consistent with this model. Nonetheless, our experiments do not rule out the alternative possibility that radiation-dependent breakdown of the intestinal barrier resulting in bacterial leakage and septicemia also plays a role in activating the innate immune response. However, studies demonstrating that innate immunity in the brain is also activated by neural injury in a fly model of traumatic brain injury (TBI) and that feeding injured flies on antibiotics does not prevent this response indicates that activation of the innate immune response in the brain can occur with neural damage independently of septicemia (Katzenberger *et al.* 2013; Katzenberger *et al.* 2015). Thus, if septicemia does contribute to induction of the innate immune response following radiation exposure, it is likely to be in addition to direct activation of inflammatory pathways by radiation. Further investigation will be necessary to determine the mechanisms by which radiation activates the immune response and the relative contributions of each pathway of activation. These experiments will have important therapeutic implications in the development of strategies to reduce the side effects of radiation therapy. It will be of interest to dissect the radiosensitive inflammatory pathway to gain a better understanding of the mechanisms that contribute to the long-term elevation in innate immune system activation. An understanding of when and how innate immune responses exert their effect following radiation exposure will be especially important for patients undergoing radiotherapy. To mitigate the deleterious consequences of sustained inflammation, therapeutic interventions to inhibit the innate immune response will need to be applied after the potential biological benefit of acute activation of the innate immune response has occurred.

Data from a number of sources have contributed to the increasing recognition of the important effects of inflammation in humans following radiation exposure. Decades after radiation exposure, atomic bomb survivors still exhibit elevated levels of inflammatory markers (Neriishi *et al.* 2001; Hayashi *et al.* 2005; Hayashi *et al.* 2012). Moreover, these survivors have an increased incidence of not only cancer but also inflammatory diseases such as liver cirrhosis and thyroid disease (Yamada *et al.* 2004). Many studies of neurodegenerative diseases have demonstrated that chronic inflammation contributes to disease pathogenesis. A better understanding of the inflammatory response to radiation will aid in the development of therapeutic strategies to mitigate the side effects of radiation exposure in the treatment of malignancies.

Work in *Drosophila* has shown that persistent overexpression of AMPs in neurons and glia can have a direct causative role in neurodegeneration (Cao *et al.* 2013; Petersen *et al.* 2012). In our *Drosophila* model of radiation-induced neurotoxicity, we observe premature aging, impaired locomotor behavior, and persistent cell death in the brains of adult flies that were irradiated during larval development (Sudmeier *et al.* 2015). Data presented here demonstrate that the innate immune system in *Drosophila* is activated by radiation exposure during larval development and remains activated following metamorphosis for at least 3 wk after radiation exposure. We hypothesize that this persistent inflammation contributes to the neurological deficits we observe in adult flies following larval irradiation. To test this hypothesis, future studies should examine whether pharmacological or genetic inhibition of the innate immune response in adults can rescue these phenotypes.

Although radiation-induced inflammation has been studied in mammalian models, flies offer a number of advantages to further our understanding of acute inflammation following radiation and the long-term consequences of sustained inflammation. Because flies lack an adaptive immune system, innate immunity can be investigated without confounding inputs from the adaptive immune system. However, the most important benefit of a fly model is likely to be application of genetic strategies to dissect the biological pathways that contribute to the effect of innate immunity on radiation-sensitive phenotypes. Our results demonstrate that the innate immune system is activated in the adult *Drosophila* brain following radiation exposure during larval development and lays the foundation for future studies of radiation-induced activation of the innate immune system in flies. These studies may facilitate the development of therapeutic strategies to reduce the deleterious side effects of radiation therapy.

## 

## Supplementary Material

Supporting Information

## References

[bib1] Armstrong G. T. Liu Q. Yasui Y. Huang S. Ness K. K., 2009 Long-term outcomes among adult survivors of childhood central nervous system malignancies in the Childhood Cancer Survivor Study. J. Natl. Cancer Inst. 101: 946–958. 19535780 10.1093/jnci/djp148 PMC2704230

[bib2] Belvin M. P. Anderson K. V., 1996 A conserved signaling pathway: the Drosophila toll-dorsal pathway. Annu. Rev. Cell Dev. Biol. 12: 393–416. 8970732 10.1146/annurev.cellbio.12.1.393

[bib3] Bulet P. Stöcklin R. Menin L., 2004 Anti-microbial peptides: from invertebrates to vertebrates. Immunol. Rev. 198: 169–184. 15199962 10.1111/j.0105-2896.2004.0124.x

[bib4] Burdelya L. G. Krivokrysenko V. I. Tallant T. C. Strom E. Gleiberman A. S., 2008 An agonist of toll-like receptor 5 has radioprotective activity in mouse and primate models. Science 320: 226–230. 18403709 10.1126/science.1154986 PMC4322935

[bib5] Cao Y. Chtarbanova S. Petersen A. J. Ganetzky B., 2013 Dnr1 mutations cause neurodegeneration in Drosophila by activating the innate immune response in the brain. Proc. Natl. Acad. Sci. USA 110: E1752–E1760. 23613578 10.1073/pnas.1306220110 PMC3651420

[bib6] Chang C. T. Lin H. Ho T. Y. Li C. C. Lo H. Y., 2011 Comprehensive assessment of host responses to ionizing radiation by nuclear factor-κB bioluminescence imaging-guided transcriptomic analysis. PLoS One 6: e23682. 21887294 10.1371/journal.pone.0023682 PMC3161058

[bib7] Chiang C. S. McBride W. H. Withers H. R., 1993 Radiation-induced astrocytic and microglial responses in mouse brain. Radiother. Oncol. 29: 60–68. 8295989 10.1016/0167-8140(93)90174-7

[bib8] Edelstein K. Spiegler B. J. Fung S. Panzarella T. Mabbott D. J., 2011 a Early aging in adult survivors of childhood medulloblastoma: long-term neurocognitive, functional, and physical outcomes. Neuro-oncol. 13: 536–545. 21367970 10.1093/neuonc/nor015 PMC3093335

[bib9] Edelstein K. D’Agostino N. Bernstein L. J. Nathan P. C. Greenberg M. L., 2011 b Long-term neurocognitive outcomes in young adult survivors of childhood acute lymphoblastic leukemia. J. Pediatr. Hematol. Oncol. 33: 450–458. 21646917 10.1097/MPH.0b013e31820d86f2

[bib10] Ellenberg L. Liu Q. Gioia G. Yasui Y. Packer R. J., 2009 Neurocognitive status in long-term survivors of childhood CNS malignancies: a report from the Childhood Cancer Survivor Study. Neuropsychology 23: 705–717. 19899829 10.1037/a0016674 PMC2796110

[bib12] Hayashi T. Morishita Y. Kubo Y. Kusunoki Y. Hayashi I., 2005 Long-term effects of radiation dose on inflammatory markers in atomic bomb survivors. Am. J. Med. 118: 83–86. 15639214 10.1016/j.amjmed.2004.06.045

[bib13] Hayashi T. Morishita Y. Khattree R. Misumi M. Sasaki K., 2012 Evaluation of systemic markers of inflammation in atomic-bomb survivors with special reference to radiation and age effects. FASEB J. 26: 4765–4773. 22872680 10.1096/fj.12-215228 PMC3475247

[bib14] Hong J. H. Chiang C. S. Campbell I. L. Sun J. R. Withers H. R., 1995 Induction of acute phase gene expression by brain irradiation. Int. J. Radiat. Oncol. Biol. Phys. 33: 619–626. 7558951 10.1016/0360-3016(95)00279-8

[bib15] Hu Z. Xing Y. Qian Y. Chen X. Tu J., 2013 Anti-radiation damage effect of polyethylenimine as a toll-like receptor 5 targeted agonist. J. Radiat. Res. (Tokyo) 54: 243–250. 23104900 10.1093/jrr/rrs098 PMC3589936

[bib16] Huttenlocher P. R., 2002 Neural Plasticity, Harvard University Press, Cambridge, Massachusetts.

[bib17] Jounai N. Kobiyama K. Takeshita F. Ishii K. J., 2012 Recognition of damage-associated molecular patterns related to nucleic acids during inflammation and vaccination. Front. Cell. Infect. Microbiol. 2: 168. 23316484 10.3389/fcimb.2012.00168 PMC3539075

[bib18] Katzenberger R. J. Loewen C. A. Wassarman D. R. Petersen A. J. Ganetzky B., 2013 A Drosophila model of closed head traumatic brain injury. Proc. Natl. Acad. Sci. USA 110: E4152–E4159. 24127584 10.1073/pnas.1316895110 PMC3816429

[bib19] Katzenberger R. J. Chtarbanova S. Rimkus S. A. Fischer J. A. Kaur G., 2015 Death following traumatic brain injury in Drosophila is associated with intestinal barrier dysfunction. Elife Mar 5: 4. 10.7554/eLife.04790 PMC4377547 25742603

[bib20] Lemaitre B. Hoffmann J., 2007 The host defense of Drosophila melanogaster. Annu. Rev. Immunol. 25: 697–743. 17201680 10.1146/annurev.immunol.25.022106.141615

[bib21] Mettler F. A. Voelz G. L., 2002 Major radiation exposure–what to expect and how to respond. N. Engl. J. Med. 346: 1554–1561. 12015396 10.1056/NEJMra000365

[bib22] Miyamoto S., 2011 Nuclear initiated NF-κB signaling: NEMO and ATM take center stage. Cell Res. 21: 116–130. 21187855 10.1038/cr.2010.179 PMC3193401

[bib23] Moding E. J. Kastan M. B. Kirsch D. G., 2013 Strategies for optimizing the response of cancer and normal tissues to radiation. Nat. Rev. Drug Discov. 12: 526–542. 23812271 10.1038/nrd4003 PMC3906736

[bib25] Mulhern R. K. Palmer S. L. Merchant T. E. Wallace D. Kocak M., 2005 Neurocognitive consequences of risk-adapted therapy for childhood medulloblastoma. J. Clin. Oncol. 23: 5511–5519. 16110011 10.1200/JCO.2005.00.703

[bib26] Myllymäki H. Valanne S. Rämet M., 2014 The Drosophila imd signaling pathway. J. Immunol. 192: 3455–3462. 24706930 10.4049/jimmunol.1303309

[bib27] Neriishi K. Nakashima E. Delongchamp R. R., 2001 Persistent subclinical inflammation among A-bomb survivors. Int. J. Radiat. Biol. 77: 475–482. 11304439 10.1080/09553000010024911

[bib28] Packer R. J. Gurney J. G. Punyko J. A. Donaldson S. S. Inskip P. D., 2003 Long-term neurologic and neurosensory sequelae in Adult survivors of a childhood brain tumor: Childhood Cancer Survivor Study. J. Clin. Oncol. 21: 3255–3261. 12947060 10.1200/JCO.2003.01.202

[bib29] Petersen A. J. Rimkus S. A. Wassarman D. A., 2012 ATM kinase inhibition in glial cells activates the innate immune response and causes neurodegeneration in Drosophila. Proc. Natl. Acad. Sci. USA 109: E656–E664. 22355133 10.1073/pnas.1110470109 PMC3306708

[bib30] Raju U. Gumin G. J. Noel F. Tofilon P. J., 1998 IkappaBalpha degradation is not a requirement for the X-ray-induced activation of nuclear factor kappaB in normal rat astrocytes and human brain tumour cells. Int. J. Radiat. Biol. 74: 617–624. 9848280 10.1080/095530098141195

[bib31] Redmond K. J. Mahone E. M. Terezakis S. Ishaq O. Ford E., 2013 Association between radiation dose to neuronal progenitor cell niches and temporal lobes and performance on neuropsychological testing in children: a prospective study. Neuro-oncol. 15: 360–369. 23322748 10.1093/neuonc/nos303 PMC3578483

[bib32] Rubin P. Johnston C. J. Williams J. P. McDonald S. Finkelstein J. N., 1995 A perpetual cascade of cytokines postirradiation leads to pulmonary fibrosis. Int. J. Radiat. Oncol. Biol. Phys. 33: 99–109. 7642437 10.1016/0360-3016(95)00095-G

[bib33] Schuitema I. Deprez S. Van Hecke W. Daams M. Uyttebroeck A., 2013 Accelerated aging, decreased white matter integrity, and associated neuropsychological dysfunction 25 years after pediatric lymphoid malignancies. J. Clin. Oncol. 31: 3378–3388. 23960182 10.1200/JCO.2012.46.7050

[bib34] Shakhov A. N. Singh V. K. Bone F. Cheney A. Kononov Y., 2012 Prevention and mitigation of acute radiation syndrome in mice by synthetic lipopeptide agonists of Toll-like receptor 2 (TLR2). PLoS One 7: e33044. 22479357 10.1371/journal.pone.0033044 PMC3314012

[bib35] Sudmeier L. J. Howard S. P. Ganetzky B., 2015 A Drosophila model to investigate the neurotoxic side effects of radiation exposure. Dis. Model. Mech. 8: 669–677. 26092528 10.1242/dmm.019786 PMC4486860

[bib36] Tauszig-Delamasure S. Bilak H. Capovilla M. Hoffmann J. A. Imler J. L., 2002 Drosophila MyD88 is required for the response to fungal and Gram-positive bacterial infections. Nat. Immunol. 3: 91–97. 11743586 10.1038/ni747

[bib38] Tzou P. Ohresser S. Ferrandon D. Capovilla M. Reichhart J. M., 2000 Tissue-specific inducible expression of antimicrobial peptide genes in Drosophila surface epithelia. Immunity 13: 737–748. 11114385 10.1016/s1074-7613(00)00072-8

[bib39] Valanne S. Wang J. H. Rämet M., 2011 The Drosophila Toll signaling pathway. J. Immunol. 186: 649–656. 21209287 10.4049/jimmunol.1002302

[bib40] Wang Y. Meng A. Lang H. Brown S. A. Konopa J. L., 2004 Activation of nuclear factor kappaB In vivo selectively protects the murine small intestine against ionizing radiation-induced damage. Cancer Res. 64: 6240–6246. 15342410 10.1158/0008-5472.CAN-04-0591

[bib41] Weiss J. F. Landauer M. R., 2003 Protection against ionizing radiation by antioxidant nutrients and phytochemicals. Toxicology 189: 1–20. 12821279 10.1016/s0300-483x(03)00149-5

[bib42] Yamada M. Wong F. L. Fujiwara S. Akahoshi M. Suzuki G., 2004 Noncancer disease incidence in atomic bomb survivors, 1958–1998. Radiat. Res. 161: 622–632. 15161358 10.1667/rr3183

[bib43] Zhou D. Brown S. A. Yu T. Chen G. Barve S., 1999 A high dose of ionizing radiation induces tissue-specific activation of nuclear factor-kappaB in vivo. Radiat. Res. 151: 703–709. 10360790

