# Supplementary material for: Persistent Activation of the Innate Immune Response in Adult Drosophila Following Radiation Exposure During Larval Development
Source: G3 (Bethesda). 2015 Sep 1;5(11):2299–306. doi: 10.1534/g3.115.021782 (PMC4632050; doi:10.1534/g3.115.021782)
Supplement: Supporting Information [file supp_5_11_2299__index.html]

Persistent Activation of the Innate Immune Response in Adult Drosophila Following Radiation Exposure During Larval Development — Supporting Information 

# Persistent Activation of the Innate Immune Response in Adult *Drosophila* Following Radiation Exposure During Larval Development

## Supporting Information for Sudmeier *et al.*, 2015

**Files in this Data Supplement:**

- Supporting Information - Tables S1-S2 (PDF, 427 KB)
- Table S1 - Primers used for qRT-PCR. (PDF, 196 KB)
- Table S2 - AMP expression in pupae 5 hours after irradiation. (PDF, 203 KB)
