# Supplementary material for: Persistent Activation of the Innate Immune Response in Adult Drosophila Following Radiation Exposure During Larval Development
Source: G3 (Bethesda). 2015 Sep 1;5(11):2299–306. doi: 10.1534/g3.115.021782 (PMC4632050; doi:10.1534/g3.115.021782)
Supplement: Supporting Information [file supp_g3.115.021782_021782SI.pdf]

G3 · Genes | Genomes | Genetics

Persistent activation of the innate immune response in adult *Drosophila* following radiation exposure during larval development

Lisa J. Sudmeier, Sai-Suma Samudrala, Steven P. Howard and Barry Ganetzky

Medical Scientist Training Program (LS)

Neuroscience Training Program (LS)

Laboratory of Genetics, University of Wisconsin-Madison, 425 Henry Mall Madison, WI 53706 (L. J. S., S.S., B. G.)

Department of Human Oncology, University of Wisconsin School of Medicine and Public Health, 600 Highland Ave. Madison, WI 53792 (S. P. H.)

Corresponding Author:

Barry Ganetzky

ganetzky@wisc.edu

Tel: 608-263-2404

425 Henry Mall

Madison, WI 53706

DOI: 10.1534/g3.115.021782

Table S1. Primers used for qRT-PCR

Primers used for qRT-PCR	5' -> 3'
Rp49 Forward	GACGCTTCAAGGGACAGTATCTG
Rp49 Reverse	AAACGCGGTTCTGCATGAG
Drosomycin (Drs) Fw	CGTGAGAACCTTTTCCAATATGATG
Drosomycin (Drs) Rv	TCCCAGGACCACCAGCAT
Drosocin (DroA) Fw	CACCCATGGCAAAAACGC
Drosocin (DroA) Rv	TGAAGTTCACCATCGTTTTCCTG
Diptericin (Dipt) Fw	GCTGCGCAATCGCTTCTACT
Diptericin (Dipt) Rv	TGGTGGAGTGGGCTTCATG
Attacin (AttC) Fw	TGGGCTACAACAATCATGGA
Attacin (AttC) Rv	GCGTATGGGTTTTGGTCAGT
Cecropin (Cec) Fw	ACGCGTTGGTCAGCACACT
Cecropin (Cec) Rv	ACATTGGCGGCTTGTTGAG
Metchnikowin (Mtk) Fw	CGTCACCAGGGACCCATTT
Metchnikowin (Mtk) Rv	CCGGTCTTGGTTGGTTAGGA

Table S2. AMP expression in pupae 5 hours after irradiation

	Drs	DroA	Dipt	AttC	Cec	Mtk
10 Gy	1.5 ± 0.9	0.7 ± 0.5	0.8 ± 0.6	0.7 ± 0.3	0.6 ± 0.1	1.1 ± 0.8
20 Gy	1.3 ± 0.4	0.4 ± 0.2	0.4 ± 0.1	0.6 ± 0.1	0.6 ± 0.1	0.5 ± 0.2
30 Gy	1.2 ± 0.5	0.6 ± 0.3	0.6 ± 0.3	0.9 ± 0.5	1.1 ± 0.9	0.9 ± 0.6
40 Gy	0.8 ± 0.3	0.5 ± 0.2	1.2 ± 0.4	0.8 ± 0.2	0.3 ± 0.1	1.2 ± 0.5

Table S2. The innate immune response is not activated in pupae 4 hours after irradiating late third instar larvae. Late third instar larvae were irradiated at the indicated doses and mRNA levels for six different AMPs were quantified in whole pupae 4-4.5 hours after irradiation. Wild-type Canton-S male and female larvae were used for these experiments. Values are given as mean expression of each AMP RNA (2-4 trials each) normalized to mean expression of the corresponding RNA in non-irradiated, age-matched controls (fold induction) ± SEM. Rp49 was used as the reference gene. See Table S1 for primers used.
