# Supplementary material for: Persistent Activation of the Innate Immune Response in Adult Drosophila Following Radiation Exposure During Larval Development
Source: G3 (Bethesda). 2015 Sep 1;5(11):2299–306. doi: 10.1534/g3.115.021782 (PMC4632050; doi:10.1534/g3.115.021782)
Supplement: Supporting Information [file supp_g3.115.021782_TableS1.pdf]

Table S1. Primers used for qRT-PCR

Primers used for qRT-PCR	5' -> 3'
Rp49 Forward	GACGCTTCAAGGGACAGTATCTG
Rp49 Reverse	AAACGCGGTTCTGCATGAG
Drosomycin (Drs) Fw	CGTGAGAACCTTTTCCAATATGATG
Drosomycin (Drs) Rv	TCCCAGGACCACCAGCAT
Drosocin (DroA) Fw	CACCCATGGCAAAAACGC
Drosocin (DroA) Rv	TGAAGTTCACCATCGTTTTCCTG
Diptericin (Dipt) Fw	GCTGCGCAATCGCTTCTACT
Diptericin (Dipt) Rv	TGGTGGAGTGGGCTTCATG
Attacin (AttC) Fw	TGGGCTACAACAATCATGGA
Attacin (AttC) Rv	GCGTATGGGTTTTGGTCAGT
Cecropin (Cec) Fw	ACGCGTTGGTCAGCACACT
Cecropin (Cec) Rv	ACATTGGCGGCTTGTTGAG
Metchnikowin (Mtk) Fw	CGTCACCAGGGACCCATTT
Metchnikowin (Mtk) Rv	CCGGTCTTGGTTGGTTAGGA
