# Supplementary material for: Persistent Activation of the Innate Immune Response in Adult Drosophila Following Radiation Exposure During Larval Development
Source: G3 (Bethesda). 2015 Sep 1;5(11):2299–306. doi: 10.1534/g3.115.021782 (PMC4632050; doi:10.1534/g3.115.021782)
Supplement: Supporting Information [file supp_g3.115.021782_TableS2.pdf]

Table S2. AMP expression in pupae 5 hours after irradiation

	Drs	DroA	Dipt	AttC	Cec	Mtk
10 Gy	1.5 ± 0.9	0.7 ± 0.5	0.8 ± 0.6	0.7 ± 0.3	0.6 ± 0.1	1.1 ± 0.8
20 Gy	1.3 ± 0.4	0.4 ± 0.2	0.4 ± 0.1	0.6 ± 0.1	0.6 ± 0.1	0.5 ± 0.2
30 Gy	1.2 ± 0.5	0.6 ± 0.3	0.6 ± 0.3	0.9 ± 0.5	1.1 ± 0.9	0.9 ± 0.6
40 Gy	0.8 ± 0.3	0.5 ± 0.2	1.2 ± 0.4	0.8 ± 0.2	0.3 ± 0.1	1.2 ± 0.5

Table S2. The innate immune response is not activated in pupae 4 hours after irradiating late third instar larvae. Late third instar larvae were irradiated at the indicated doses and mRNA levels for six different AMPs were quantified in whole pupae 4-4.5 hours after irradiation. Wild-type Canton-S male and female larvae were used for these experiments. Values are given as mean expression of each AMP RNA (2-4 trials each) normalized to mean expression of the corresponding RNA in non-irradiated, age-matched controls (fold induction) ± SEM. Rp49 was used as the reference gene. See Table S1 for primers used.
